# 14-3-3 Binding and Sumoylation Concur to the Down-Modulation of *β-catenin* Antagonist *chibby 1* in Chronic Myeloid Leukemia

**DOI:** 10.1371/journal.pone.0131074

**Published:** 2015-07-06

**Authors:** Manuela Mancini, Elisa Leo, Ken-Ichi Takemaru, Virginia Campi, Fausto Castagnetti, Simona Soverini, Caterina De Benedittis, Gianantonio Rosti, Michele Cavo, Maria Alessandra Santucci, Giovanni Martinelli

**Affiliations:** 1 Department of Experimental Diagnostic and Specialty Medicine—DIMES—Institute of Hematology "L. and A. Seràgnoli". University of Bologna-Medical School, Bologna, Italy; 2 Department of Pharmacological Sciences, State University of New York at Stony Brook, Stony Brook, New York, United States of America; Università degli Studi di Firenze, ITALY

## Abstract

The down-modulation of the β-catenin antagonist Chibby 1 (CBY1) associated with the *BCR-ABL1* fusion gene of chronic myeloid leukemia (CML) contributes to the aberrant activation of β-catenin, particularly in leukemic stem cells (LSC) resistant to tyrosine kinase (TK) inhibitors. It is, at least partly, driven by transcriptional events and gene promoter hyper-methylation. Here we demonstrate that it also arises from reduced protein stability upon binding to 14-3-3σ adapter protein. CBY1/14-3-3σ interaction in BCR-ABL1+ cells is mediated by the fusion protein TK and AKT phosphorylation of CBY1 at critical serine 20, and encompasses the 14-3-3σ binding modes I and II involved in the binding with client proteins. Moreover, it is impaired by c-Jun N-terminal kinase (JNK) phosphorylation of 14-3-3σ at serine 186, which promotes dissociation of client proteins. The ubiquitin proteasome system UPS participates in reducing stability of CBY1 bound with 14-3-3σ through enhanced SUMOylation. Our results open new routes towards the research on molecular pathways promoting the proliferative advantage of leukemic hematopoiesis over the normal counterpart.

## Introduction

Chronic myeloid leukemia (CML) is a myeloproliferative disease originated from a pluripotent hematopoietic cell, the putative leukemic stem cell (LSC), and caused by a single genetic lesion, the t(9;22)(q34;q11) reciprocal translocation. The resulting *BCR-ABL1* rearranged gene encodes a p210-kDa chimeric protein where the *ABL* tyrosine kinase (TK) is converted into a constitutively activated isoform by fusion with the *BCR* amino acids 1 to 63 [1,2]. The majority of CML patients undergo complete hematologic remission in response to TK inhibitor imatinib (IM) [3]. However, BCR-ABL1+ LSC are neither dependent on *BCR-ABL1 TK* for proliferation and survival nor killed by IM and the second generation inhibitors Nilotinib and Dasatinib, hence providing a sanctuary for disease recurrence upon drug withdrawal and a putative source of drug-resistance [4]. Signals promoting *BCR-ABL1*+ LSC survival and proliferation are therefore subjects of intensive investigation in view of their clinical relevance as pharmacological targets complementary to *BCR-ABL1* inhibition. Among them, β-catenin is crucial for self-renewal and persistence under TK inhibitor therapy of BCR-ABL1+ LSC and committed granulocyte/macrophage progenitor reprogramming into LSC at the blast crisis (BC) onset [5–8].

The activation of β-catenin in CML is driven by post-translational modifications, namely the *BCR-ABL1*-mediated phosphorylation at specific tyrosine residues (Y86 and Y654), which enhances protein stability by impairing its recruitment to the adenomatous polyposis coli (APC)/Axin/glycogen synthase kinase-3β (GSK-3β) destruction complex [9]. Moreover, it is promoted by the deregulated transcription of genes encoding factors critical for β-catenin inactivation, including growth arrest-specific 2 (GAS2, which reduces the calpain-dependent degradation of β-catenin), and by the prevalence of GSK-3β mis-spliced, functionally inactive isoforms unable to phosphorylate β-catenin and/or Fas-associated phosphatase 1 (Fap1, which inactivates GSK-3β through de-phosphorylation) [10–13]. Inhibition of β-catenin proteasomal degradation and subsequent cytoplasmic accumulation lead to its nuclear translocation and binding with the T-cell factor/lymphoid enhancer factor 1 (TCF/LEF1) transcription factors to form a transcriptionally active complex [14]. We have recently provided evidence that the *BCR-ABL1*-associated down-modulation of CBY1 also contributes to aberrant β-catenin activation in CML [15]. CBY1 is a small conserved protein that antagonizes β-catenin transcriptional activity through interaction with the β-catenin C-terminal activation domain (hampering binding of β-catenin to TCF/LEF1) and with the 14-3-3 scaffolding proteins (promoting β-catenin nuclear export) [16,17]. CBY1 down-modulation is a distinct trait of the putative LSC compartment identified by a CD34+ phenotype, contingent upon *BCR-ABL1* expression and TK activity, and associated with activation of β-catenin signaling [15]. It is, at least partly, evoked by transcriptional events driven by the gene promoter hyper-methylation [18]. The prominent reduction of CBY1 protein compared to transcript levels suggests that enhanced protein degradation may contribute to CBY1 down-modulation in CML hematopoietic progenitors [15]. Here, we investigated the molecular mechanisms underlying the reduced stability and degradation of CBY1 in association with *BCR-ABL1*. First, we demonstrated that dissociation of CBY1 from 14-3-3σ raises CBY1 protein levels by enhancing its stability. Moreover, we found that enhanced SUMOylation of CBY1 may be an important factor contributing to CBY1 degradation in CML hematopoietic progenitors.

## Materials and Methods

### Ethics statement

CML patients included in the study provided their written informed consent to be enrolled in clinical trials NCT01535391, NCT01761890, NCT01699217 and NCT 01650805 (ref clinicaltrials.gov) and be evaluated for response to therapy with imatinib, nilotinib, dasatinib or ponatinib according to the study design. The above mentioned clinical trials were approved by the Ethical Committee of the Policlinico S.Orsola-Malpighi. Patients gave written consent to the use of their bone marrow samples for genetic and laboratory biomarker analyses. Moreover, they gave verbal informed consent to participate in this specific study at the moment of their enrollment in the clinical trials, according to the guidelines of the Ethical Committee of the Policlinico S.Orsola-Malpighi. The verbal informed consent was properly registered in the medical record. Once collected, all samples were given an anonymous code and stored following the recommendations of aforementioned trials. Samples from 8 healthy donor (HD) subjects were collected at the moment of bone marrow harvest intended for bone marrow transplantation. Participants were verbally informed and consented to the use of residual fractions of the harvested samples for this study. The verbal consent was registered in their medical records according to the consent procedures approved by the Ethical Committee of the Policlinico S.Orsola-Malpighi. All methods employed in this study were in accordance with the Declaration of Helsinki.

### Study population

Twelve CML-CP patients were included in our study. Clinical details are given in the S1 Table. All of them exhibited the BCR-ABL1 rearranged gene coding for p210-kDa fusion protein. The mononuclear cell fraction (MCF) from bone marrow samples of patients and healthy donors (HD) were obtained by means of Ficoll-Hypaque gradient.

Equal amounts of proteins from bone marrow samples of 8 HD were pooled to avoid individual differences in protein expression. SDS-PAGE signal intensities of the HD pool were normalized to 1 and kept as reference values of signal intensities in CML-CP patients. Informed consent to report their clinical details and results of biomolecular analyses was preliminarily obtained according to protocols NCT01535391, NCT01761890, NCT01699217 and NCT 01650805 (see the above section for details).

### Cells and treatments

A construct containing the whole wild-type (WT) CBY1 coding sequence was obtained through amplification of cDNA from the HepG2 cell line and inserted into a commercial plasmid (pcDNA3.1, Invitrogen) containing the neomycin phosphotransferase gene, which allows cell selection in RPMI 1640 supplemented with the neomycin analog G418 [19]. WT CBY1 construct was transfected in the BCR-ABL1+ cell line K562 by means of electroporation at 0.25V/960 mF (Equibio Easyject, Optima). Its stable expression in transfected cells was achieved after two month selection in RPMI 1640 medium (Lonza) supplemented with 10% fetal calf serum (FCS, Gibco), 1% L-Glutamine, antibiotics and 500 μg/mL G418 in 5% CO_2_ and fully humidified atmosphere at 37°C [19]. Parental K562 is a BCR-ABL1+ cell line that exhibits low transcript levels of CBY1 and undetectable protein level. IM (2 μM) was used to inhibit BCR-ABL1 TK activity in parental and wt CBY1-transfected K562 cells. RAD001 (100 nM) was used to inhibit mTOR to investigate the impact of AKT activity on CBY1 stability. BV02 (5 μM) was used to inhibit 14-3-3 binding to CBY1 to investigate its role in CBY1 degradation. SP600125 (5 μM) was used to inhibit JNK kinase activity. Finally, Bortezomib (5 nM) was used as proteasome inhibitor, to evaluate if CBY1 degradation was a proteasome-dependent or independent mechanism.

Apoptotic cell death was measured by the uptake of fluorescinated Annexin V and propidium iodide (PI, both from Roche) using a FACsCantoII flow cytometer (Beckton Dickinson) set at 488 nm excitation and 530 nm wavelength bandpass filter for fluorescin detection or 580 nm for PI detection, and a dedicated software (DIVA software, Beckton Dickinson).

### Protein analysis

Western blot (WB) and immunoprecipitation (IP)/immunoblotting analyses were performed on whole cell lysates and nuclear fractions according to published methods [19,20]. The anti-phospho-c-Abl (Tyr245), anti-β-catenin, anti-14-3-3 (pan), anti-phospho-14-3-3 (Ser186) and anti-Sumo 1 antibodies were purchased from Cell Signaling Technology. The anti-phospho-JNK (Thr 183) was purchased from Merck Millipore. The anti-CBY1 was previously described [16]. The anti-β-actin antibody used as loading control was purchased from Santa Cruz Biotechnology. The anti-histone H1 used as control for nuclear protein loading was purchased from Genetex. Signal intensities in single blots obtained in three separate experiments were measured by means of ChemiDoc-It instrument (UVP) equipped with a dedicated software (Launch VisionWorksLS from Euroclone). The differences among signal intensities were evaluated for statistical significance using the paired Student’s *t*-test.

## Results

### BCR-ABL1 TK activity impacts on CBY1 binding with 14-3-3σ, expression and sub-cellular location

**Fig 1:**
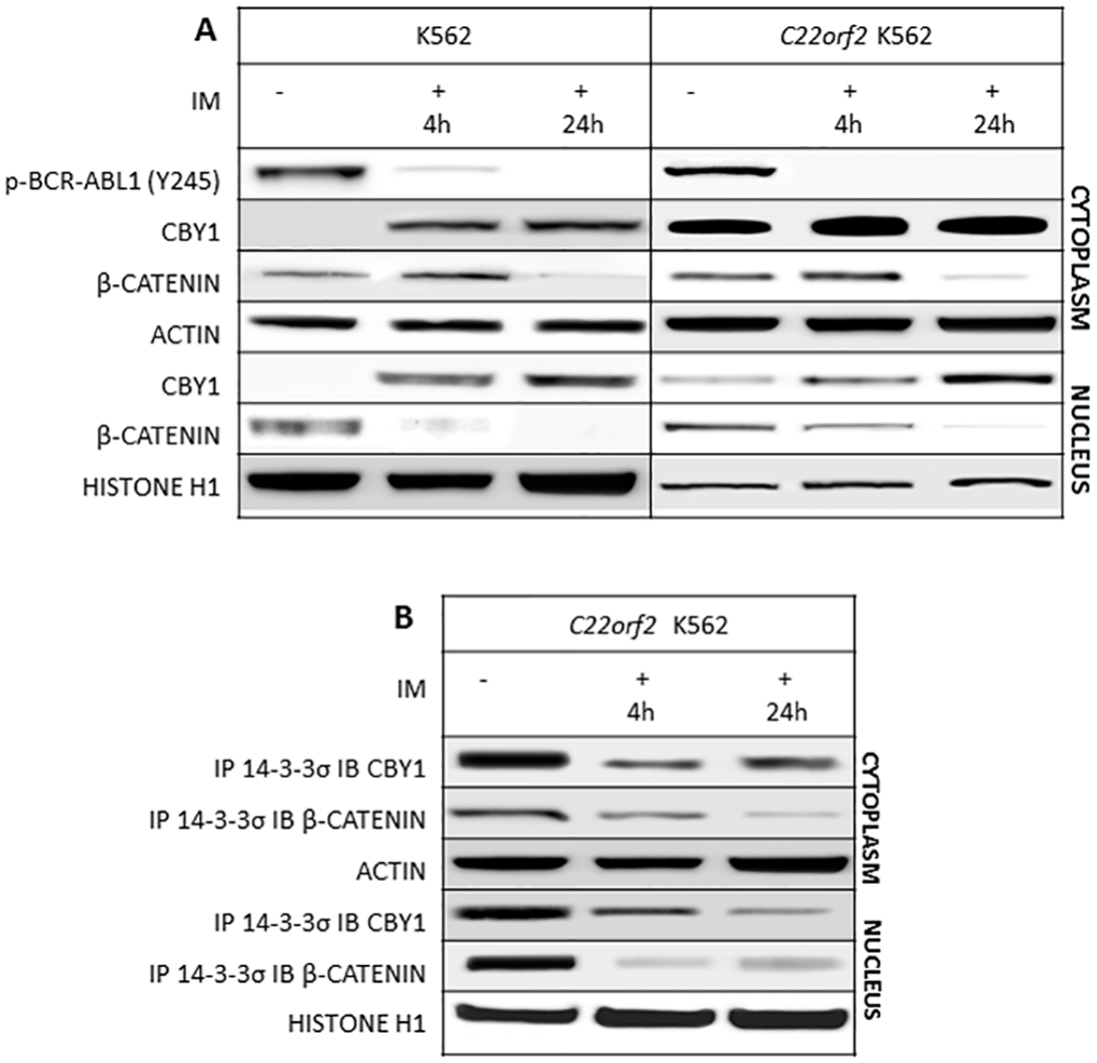
IM promotes changes in CBY1 and β-catenin expression associated with the dissolution of CBY1 and β-catenin binding with 14-3-3σ. A- Cytoplasmic and nuclear protein analysis was performed after 4 and 24 hours of exposure to IM. In first instance, *BCR-ABL1* de-phosphorylation at the critical residue for constitutive activation of the fusion protein enzymatic activity (tytosine-Y-245) was assessed, hence proving IM inhibitory effect on its target at the time other protein expression and interactions were investigated; B- WB and IP/IB have been performed according to published procedures and confirmed in three independent experiments. Densitometric analysis of signal intensities shows a statistically significance difference (p<0.05; Student’s t test) in treated vs untreated cells. Actin and histone H1 were used as control for loading of cytoplasmic and nuclear proteins, respectively. Lack of IM off target effects is shown in the Supplementary section, S2 Fig

Our previous study established that CBY1 down-modulation participates in β-catenin activation in CML [15]. CBY1 reduction is contingent upon the *BCR-ABL1* TK activity and driven by transcriptional events encompassing DNA hyper-methylation at the promoter-associated CpG islands of the CBY1-encoding gene *C22orf2* [18]. Notably, the greater reduction of CBY1 protein compared to transcript suggests that enhanced protein degradation contributes to CBY1 down-modulation in CML hematopoietic progenitors [15]. Previous studies underscored that CBY1 has a central role in β-catenin nuclear export, contingent upon its binding with 14-3-3σ and ξ scaffolding proteins in a stable and tripartite complex encompassing β-catenin [16,17]. Here we investigated the impact of 14-3-3σ binding on CBY1 expression and stability in a *BCR-ABL1+* cell context. The study was conducted in parental K562, a *BCR-ABL1+* cell line, which exhibits low CBY1 transcript and undetectable protein levels, and in a K562 polyclonal cell population stably transfected with a *C22orf2* construct coding for the wt CBY1 (*C22orf2* K562) [19]. Due to the inherent lack of CBY1 in parental K562 cell line, most results shown here concern *C22orf2* K562, where CBY1 is over-expressed [21]. In first instance, 14-3-3σ IP products were probed with anti-CBY1 or anti-β-catenin antibody and compared for signal intensities under experimental conditions hampering their interaction with the scaffolding protein. The choice of performing IP with anti-14-3-3σ antibody was dictated by the absence of significant differences in 14-3-3σ levels in treated cells compared to untreated controls (see S1 Fig). Our previous studies suggested that reduced CBY1 expression is contingent upon the *BCR-ABL1* TK activity. A significant increase in both cytoplasmic and nuclear CBY1 levels was, in fact, seen both in parental and *C22orf2* K562 cell lines after 4 and 24 h of exposure to IM (2 μM) (p<0.05) (Fig 1A). CBY1 induction in response to IM was, at least partly, driven by enhanced transcription following gene promoter de-methylation (S2 Fig) [18]. It clearly correlated with the nuclear export of β-catenin, which is followed by β-catenin degradation and inactivation in the cytoplasm (Fig 1A) [9]. Further investigation established that 14-3-3σ binding has a role in changes of CBY1 and β-catenin expression and sub-cellular partitioning in response to IM. *BCR-ABL1* TK inactivation after 4 and 24 h of exposure to IM was, in fact, associated with a significant reduction of CBY1 and βcatenin interaction with 14-3-3σ in cytoplasmic and nuclear compartments of *C22orf2* K562 (p<0.05) (Fig 1A and 1B). Unlike CBY1, βcatenin release from 14-3-3σ in response to IM was associated with an initial significant increase (4h) in the cytoplasmic compartment, due to its nuclear export (p<0.05), followed by a complete loss, due to its degradation (Fig 1A) [9]. These findings suggest that CBY1 and βcatenin dissociation from 14-3-3σ in response to IM has opposite effects on CBY1 and βcatenin expression.

### 14-3-3 binding motifs have a central role in CBY1 interaction with 14-3-3σ, expression and sub-cellular location

**Fig 2:**
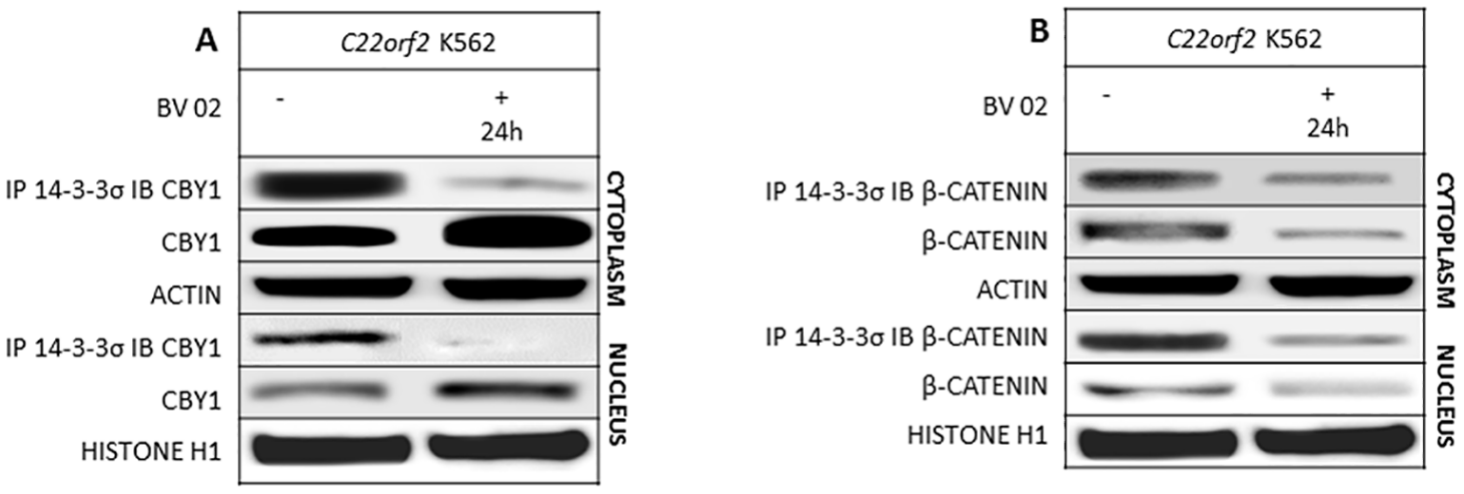
The dissolution of CBY/14-3-3σ complex in response to BV02, an inhibitor of 14-3-3 binding modes I and II, is associated with a CBY1 increment in the cytoplasmic and nuclear compartment. A- CBY1 expression and interaction with 14-3-3σ and βcatenin expression and interaction with 14-3-3σ were assayed in the cytoplasmic and nuclear compartments of *C22orf2* K562 cells at 24^th^ hour of exposure to BV02. See legend to Fig 1 for technical details.

The association of 14-3-3 with partner proteins is impaired by BV02, a small molecule inhibitor of both mode I and II 14-3-3σ-binding motifs, used to confirm the effects of 14-3-3σ binding on the expression levels and partitioning of CBY1 and βcatenin [22]. The lack of BV02 off-target effects were confirmed in *C22orf2* K562 cell line (S3 Fig). As expected, CBY1 interaction with 14-3-3σ was significantly reduced upon BV02 treatment (5 μM for 24 h) (p<0.001) in the cytoplasmic compartment of *C22orf2* K562 cells and completely abrogated in the nuclear compartment (Fig 2A). The release from 14-3-3σ was associated with a significant increase in CBY1 protein levels both in cytoplasmic and nuclear fractions (p<0.01 and p<0.05, respectively), not contingent upon transcriptional mechanisms (data not shown). These data suggest that impaired 14-3-3σ binding does not preclude the nuclear-cytoplasmic shuttling of CBY1 and is involved in CBY1 stabilization under conditions which do not affect *BCR-ABL1* TK activity. A likewise reduction of βcatenin-14-3-3σ interaction in response to BV02 was seen in cytoplasmic and nuclear compartments of *C22orf2* K562 cells (p<0.01 and p<0.05, respectively) (Fig 2B). βcatenin expression levels were significantly reduced in both compartments (p<0.01 and p<0.05, respectively) (Fig 2B). These results suggest that loss of 14-3-3σ binding does not hinder βcatenin nuclear export.

### AKT constitutive activation by BCR-ABL1 TK affects CBY1 interaction with 14-3-3σ, expression and sub-cellular location

CBY1 binding with 14-3-3 proteins σ and ξ is mediated by AKT phosphorylation at serine 20, located within the mode II 14-3-3-binding motif [17]. Notably, AKT inhibition due to inactivation of *BCR-ABL1* TK by IM is an early event, followed by its retrograde activation by mTOR [23,24]. The recovery of AKT activating phosphorylation at serine 473 after 24 h of exposure to IM was confirmed in our experimental model (S3 Fig).

**Fig 3:**
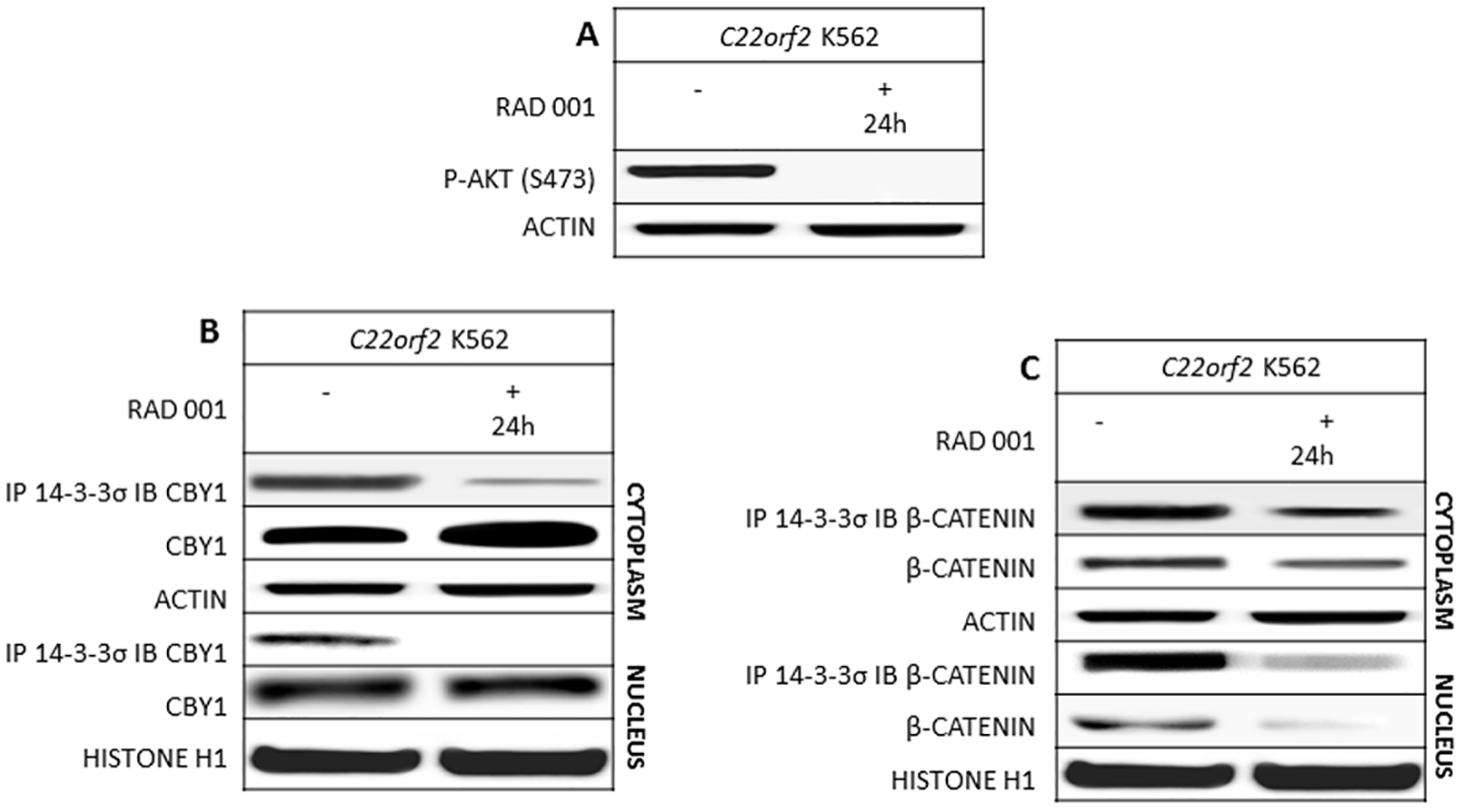
AKT inactivation in response to m-TOR inhibitor RAD001 hinders CBY1 interaction with 14-3-3σ and is associated with cytoplasmic CBY1 increment. A- RAD001 inhibits its target: AKT; B- CBY1 expression and interaction with 14-3-3σ and C- βcatenin expression and interaction with 14-3-3σ were assayed in the cytoplasmic and nuclear compartments of *C22orf2* K562 cells at 24^th^ hour of exposure to RAD001. The absence of off-target effects was assayed (Supplementary section, S2 Fig). See legend to Fig 1 for technical details.

RAD001 (everolimus), an mTOR inhibitor promoting persistent AKT inactivation, was used to investigate the impact of AKT inhibition on CBY1 and βcatenin subcellular localization and expression in relation to their interaction with 14-3-3σ [25]. The absence of RAD001 off-target effects in *C22orf2* K562 cell line was preliminary assayed (Supplementary section, S3 Fig). Persistent AKT de-phosphorylation at serine 473 upon treatment with RAD001 was associated with a significant reduction of CBY1 interaction with 14-3-3σ in the cytoplasm (p<0.01) and its complete abolition in the nuclear compartment of *C22orf2* K562 cells (Fig 3A and 3B). CBY1 release from 14-3-3σ in response to RAD001 was associated with a significant increase of CBY1 levels in the cytoplasm (p<0.01), but not in the nucleus (p<0.1) (Fig 3B). The increment of cytoplasmic CBY1 in response to RAD001 was not driven by enhanced transcription (data not shown). It might be ascribed to enhanced stability, while CBY1 steady levels in the nucleus might be attributed to impaired nuclear export due to AKT inhibition. βcatenin expression and interaction with 14-3-3σ were similarly reduced in response to RAD001 in both compartments (p<0.05), further supporting the role of 14-3-3σ binding in protecting βcatenin from degradation and preventing nuclear re-import in the presence of activated *BCR-ABL1* TK (Fig 3C).

### JNK intervenes in the cohesion of CBY1/14-3-3σ complex

**Fig 4:**
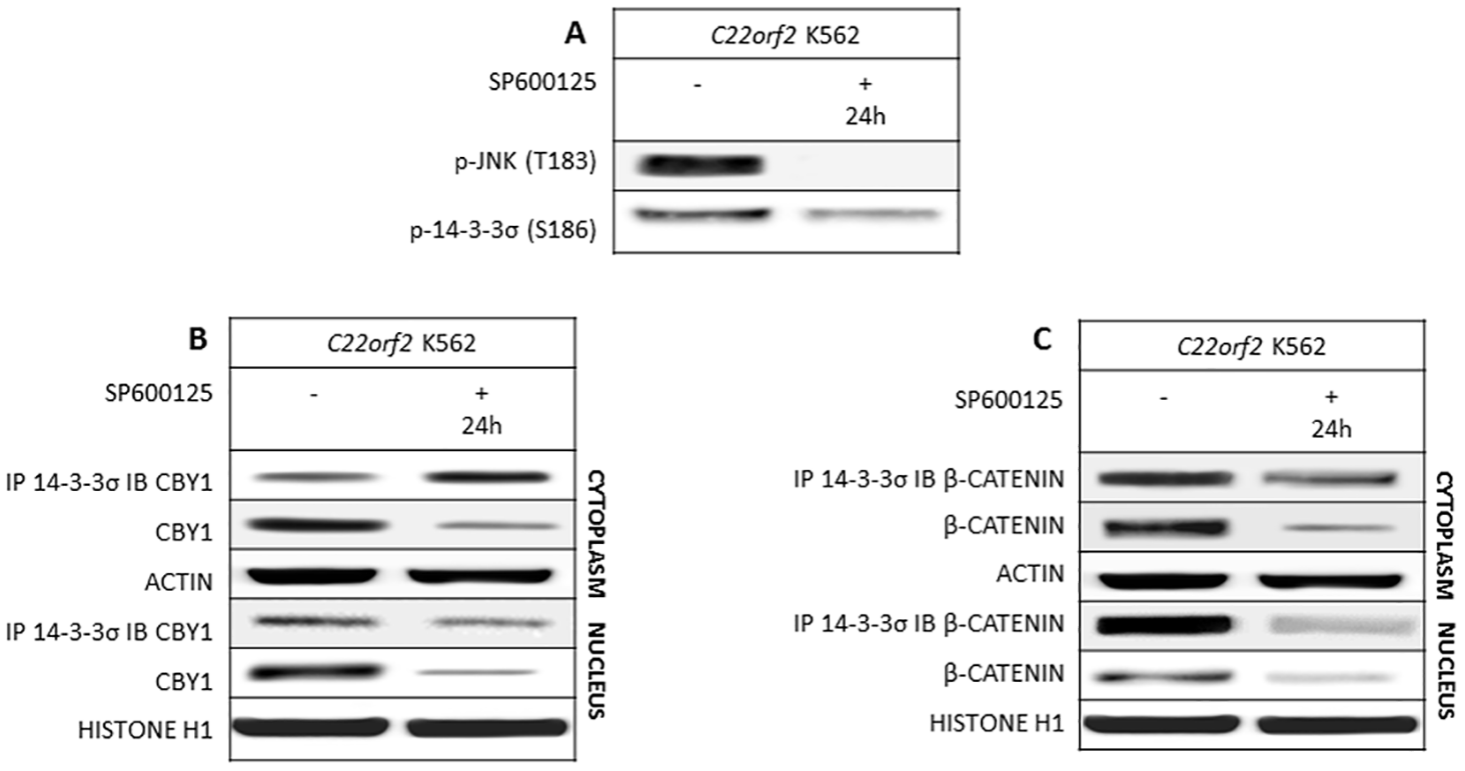
JNK and 14-3-3σ de-phosphorylation at threonine 183 and serine 186, respectively, in response to JNK inhibitor SP600125 prevents the dissolution of CBY1/14-3-3σ complex and leaves steady the cytoplasmic expression of CBY1. A- SP600125 inhibitory effects on its targets (JNK and 14-3-3); B- CBY1 expression and interaction with 14-3-3σ and βcatenin expression and interaction with 14-3-3σ were assayed in the cytoplasmic and nuclear compartments of *C22orf2* K562 cells 24^th^ hour of exposure to SP600125. The absence of off-target effects was assayed (Supplementary section, S2 Fig). See legend to Fig 1 for technical details.

The binding of 14-3-3 with client proteins is further regulated by their own post-translational modifications. In particular, phosphorylation of 14-3-3σ and ξ at discrete sites (serine 186 and 184, respectively) by c-Jun N-terminal kinase (JNK) promotes the dissociation of client proteins [26]. The impact of JNK inhibitor SP600125 (25 μM for 24 h) on JNK auto-phosphorylation at threonine 183 and 14-3-3σ phosphorylation at serine 186 was preliminarily evaluated (Fig 4A). Moreover, SP600125 off-target effects on other signaling components critical for interactions of CBY1 and βcatenin with 14-3-3σ were excluded (S3 Fig). The inhibition of JNK-induced phosphorylation of 14-3-3σ in response to SP6000125 significantly enhanced CBY1/14-3-3σ interaction in cytoplasmic compartment of *C22orf2* K562 (<0.01) and left steady CBY1/14-3-3σ binding in the nuclear compartment (p<0.1) (Fig 4B). Further investigation is required to elucidate the causes of such difference. In all instance, persistent CBY1 interaction with 14-3-3σ was associated with a significant reduction in CBY1 expression in both compartments of *C22orf2* K562 cells (p<0.01) (Fig 4B). These findings support that JNK de-phosphorylation prevents CBY1 dissociation from 14-3-3σ by hindering 14-3-3σ post-translational events critical for client protein release. Persistent CBY1/14-3-3σ interaction was associated with cytoplasmic and nuclear CBY1 reduction, further supporting the hypothesis raised by previous results that the interaction with 14-3-3 impacts CBY1 stability. Conversely, βcatenin/14-3-3σ interaction in the cytoplasmic and nuclear compartment of *C22orf2* K562 cells was impaired by SP600125 (p<0.05 and p<0.01, respectively) (Fig 4C). These findings suggest that JNK/14-3-3σ axis is not critical to keep βcatenin bound with 14-3-3σ and that JNK might rather induce βcatenin post-transcriptional modifications at critical residues for binding with 14-3-3σ [27,28]. Moreover, βcatenin expression was significantly reduced in both compartments in response to SP600125 (p< 0.01), further supporting that the release from 14-3-3σ directs cytoplasmic βcatenin towards degradation, concurrently precluding βcatenin nuclear import even in the presence of activated *BCR-ABL1* TK. CBY1 increment in response to IM, BV02 and RAD001 and CBY1 absence upon treatment with SP600125 were confirmed in the cytoplasm of parental K562 cell line (S4 Fig).

### CBY1 reduced expression in leukemic hematopoiesis is associated with JNK and 14-3-3σ reduced phosphorylation and CBY1 enhanced interaction with 14-3-3σ

**Fig 5:**
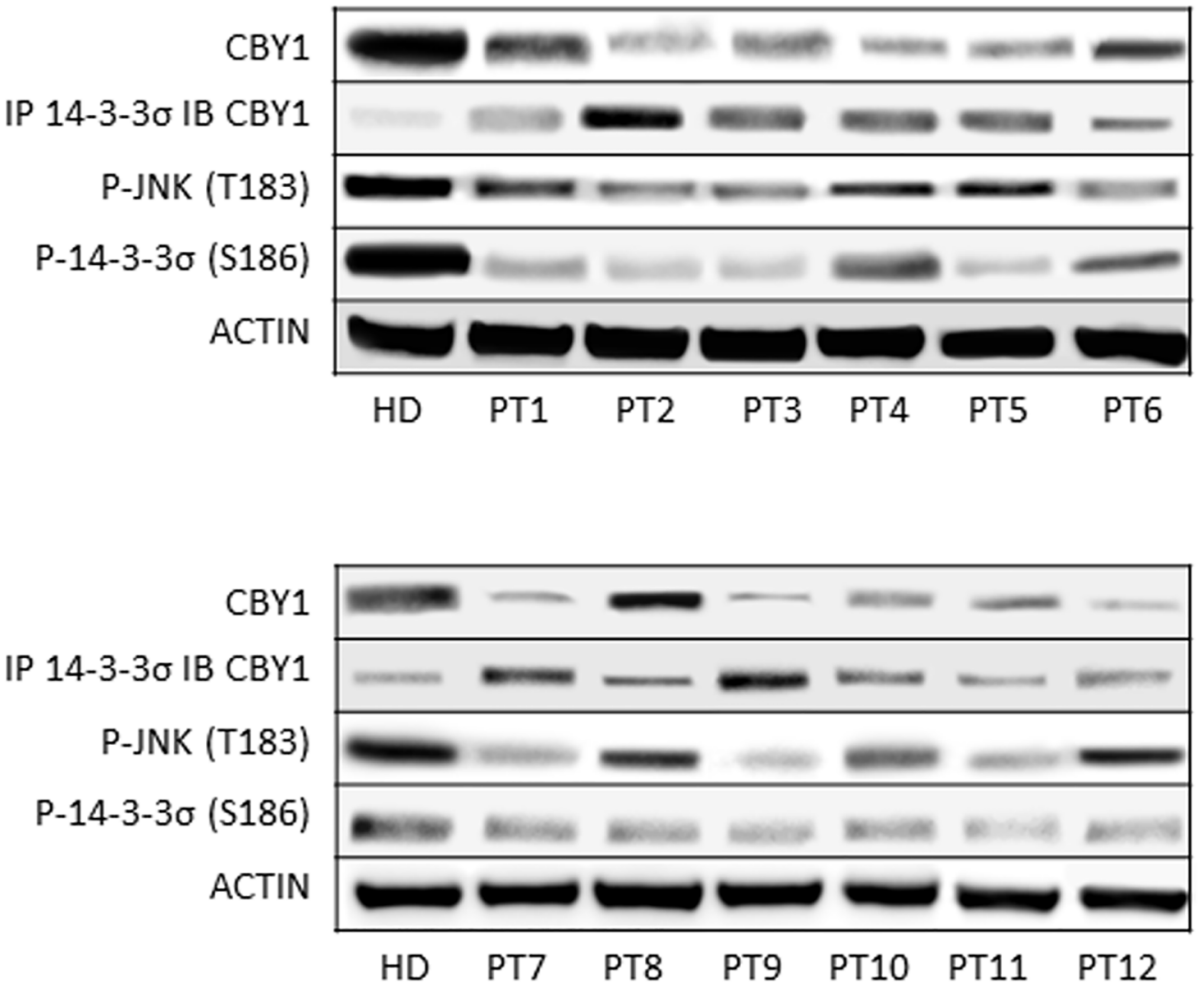
Reduction of JNK and 14-3-3σ post-translational modifications is associated with CBY1 reduced expression and interaction with 14-3-3σ in CML. Bone marrow MCF of 12 CML patients at diagnosis and 8 HDs were compared for CBY1 expression and interaction with 14-3-3σ, JNK phosphorylation at threonine 183 and 14-3-3σ phosphorylation at serine 186. Clinical details of CML patients included in our study are illustrated in the Supplementary section, S1 Table. A pool consisting of equal amounts of proteins from MCF of bone marrow samples of HDs was used as control for IP/IB to avoid individual differences. See legend to Fig 1 for technical details. The two blots shown here have been run separately and confirmed by two additional experiments.

The participation of JNK and 14-3-3σ phosphorylation in CBY1 interaction with 14-3-3σ and expression was further investigated in MCF from bone marrow samples of 12 CML patients at diagnosis and HD (pooled to avoid individual differences). Clinical characteristics of CML patients included in our study are illustrated in the S1 Table. CBY1 cytoplasmic levels were significantly lower in all patients compared to the normal control pool (p<0.05 or less), confirming our previously published results [15]. Moreover, the interaction of CBY1 with 14-3-3σ was significantly enhanced in *BCR-ABL1*+ cells compared to the HD pool (p<0.01 or less) (Fig 5). CBY1 reduced expression and enhanced binding with 14-3-3σ were associated with a significant reduction in JNK phosphorylation at threonine 183 and 14-3-3σ phosphorylation at serine 186 (p<0.05 or less) (Fig 5). These results are consistent with the impact of JNK-induced post-transcriptional modification of 14-3-3σ, in addition to AKT-induced CBY1 phosphorylation, on CBY1 interaction with 14-3-3σ and its role in CBY1 down-modulation associated with *BCR-ABL1* TK activity.

### CBY1 reduced expression associated with BCR-ABL1 TK is provoked by proteasomic degradation and mediated by the protein SUMOylation

**Fig 6:**
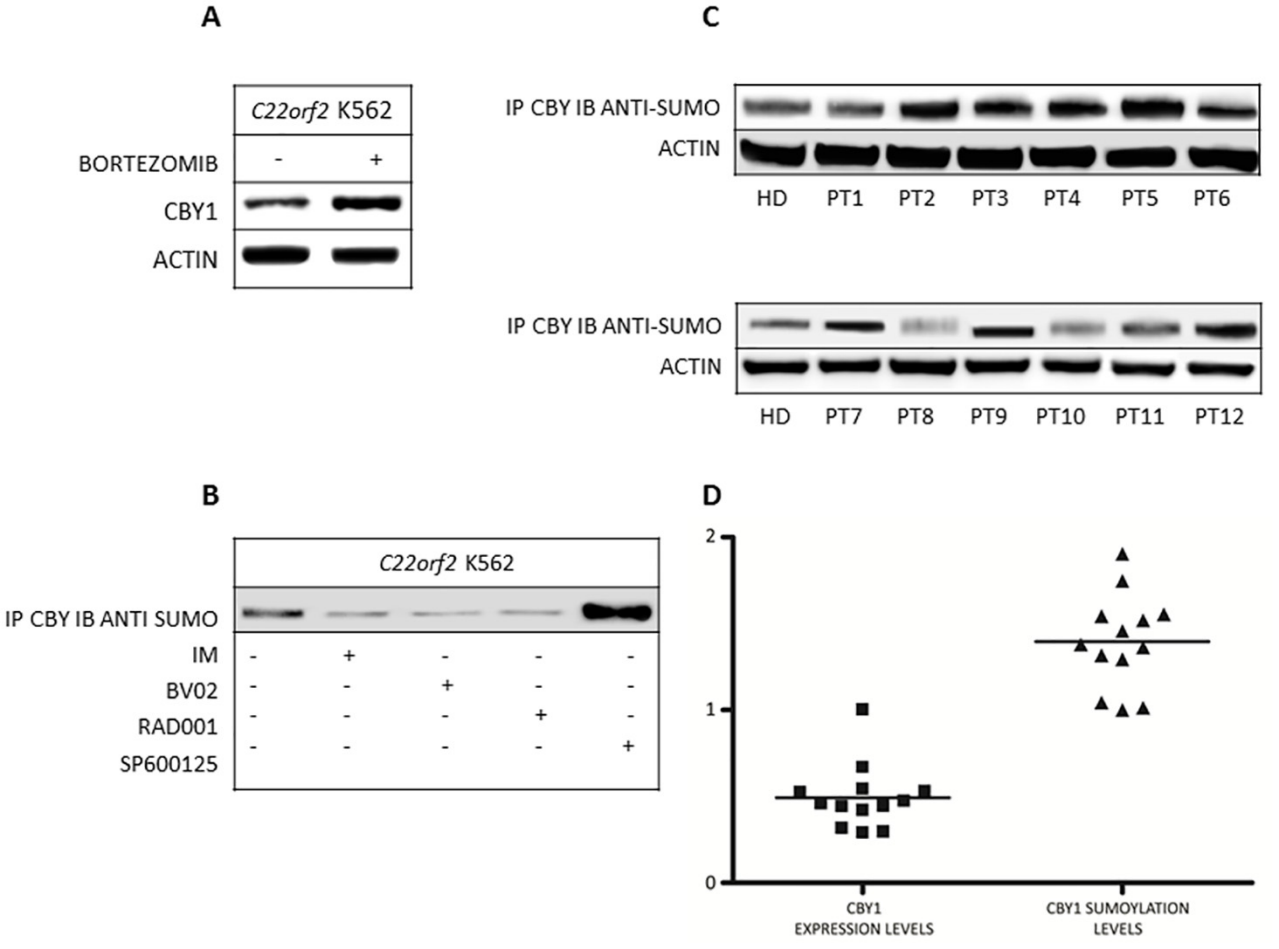
Proteasome degradation through SUMOylation has a role in CBY1 reduced stability associated with BCR-ABL1 TK-mediated binding with 14-3-3σ. A- Cytoplasmic CBY1 expression was upraised at 24th hour of treatment with Bortezomib (5nM) in *C22orf2* K562 cells; B- Reduction of CBY1 SUMOylation was associated with CBY1 upraised expression in response to compounds (IM, BV02 and RAD001) which impair the interaction with 14-3-3σ. Conversely, CBY1 SUMOylation was increased in response to the JNK inhibitor, which precludes CBY1 release from 14-3-3σ and reduces CBY1 cytoplasmic levels; C- Higher CBY1 SUMOylation was seen in MCF from bone marrow samples of 12 CML patients at diagnosis compared to pooled HD. Clinical details of CML patients included in our study are illustrated in the S1 Table. The two blots shown here have been run separately and confirmed by two additional independent experiments. D- CBY1 SUMOylation in MCF from CML patients was inversely correlated with the expression of CBY1. Signal intensities of WB obtained with equal amounts of protein from CML and HD samples. CBY1 expression exhibited a decrease of more than 50% with respect to the normal control, while SUMOylation showed an almost 1,5-fold increment compared to the normal control (signal intensity of HD protein pool was set to 1 to represent the reference value). See legend to Fig 1 for technical details.

Selective degradation of the great majority of intracellular proteins is executed by the ubiquitin proteasome system (UPS) [29–31]. Bortezomib, an N-protected dipeptide, whose boronic acid at the C-terminus binds and inhibits the catalytic site of the 26S proteasome, was used to assess whether enhanced UPS degradation is implicated in CBY1 down-modulation associated with *BCR-ABL1* [29]. In *C22orf2* K562, CBY1 exhibited a significant increment in response to bortezomib (5 nM for 24 h) (p<.001) (Fig 6A). The findings raise the possibility that UPS is a component of CBY1 down-modulation in CML. The detection of small ubiquitin-like modifier (SUMO) groups in the CBY1 sequence addressed further investigation towards SUMOylation as a mechanism involved in CBY1 increased degradation associated with *BCR-ABL1*, CBY1 SUMOylation status was investigated under experimental conditions affecting CBY1/14-3-3σ interaction used in the first part of our work. To this purpose, CBY1 IP products from *C22orf2* K562 cells treated with drugs either promoting (IM, BV02 and RAD001) or preventing (SP600125) CBY1 release from 14-3-3σ were probed with anti-SUMO antibodies. CBY1 increment following its release from 14-3-3σ in response to IM, BV02 and RAD001 was associated with a significant reduction in the signal intensities with anti-SUMO antibody (p<0.01) (Fig 6B). Conversely, the reduction of CBY1 expression associated with the persistence of CBY1/14-3-3σ upon SP600125 treatment was associated with a significant increment in the band intensities with anti-SUMO antibody (p<0.001) (Fig 6B). The findings suggest that SUMOylation may intervene in the degradation of CBY1 bound with 14-3-3σ.

To confirm that SUMOylation is a component of CBY1 down-modulation in CML, we compared signal intensities of CBY1 IP products from MCF of CML patients and HD probed with anti-SUMO antibody. In all CML patients but one (patient #1) CBY1 reduced expression was associated with a significant increment in its SUMOylation compared to the HD pool (p<0.05 or less) (Fig 6C). Densitometric analysis of signal intensities of CBY1 IP products immunoblotted with anti-CBY1 and anti-SUMO antibodies revealed the inverse correlation between CBY1 expression and CBY1 SUMOylation in patients as compared to the HD pool (Fig 6D).

## Discussion

CBY1 down-modulation is a component of βcatenin activation in CML [15]. It is partly contingent upon transcriptional events and driven by promoter hyper-methylation (S2 Fig) [18]. Results of this study reveal that it is also driven by the reduced protein stability associated with *BCR-ABL1* TK activity and by CBY1 interaction with 14-3-3σ. 14-3-3 proteins are ubiquitous adapter proteins whose physiological effects on cell cycle, signal transduction, protein trafficking and apoptosis are contingent upon binding with client proteins at a so-called amphipathic groove, which accommodates the mostly phosphorylated interaction motif of their partner proteins [32] Indeed, CBY1 binding with 14-3-3 (either σ or ξ) promotes βcatenin nuclear export in a stable tripartite complex, hence providing an alternative mechanism to competition for TCF/LEF1 binding in βcatenin transcriptional inactivation [17]. 14-3-3 binding may either enhance or inhibit the client protein activities [33]. Notably, the 14-3-3 interaction with core components of the mitochondrial apoptotic machinery, including the BCL-2 antagonist of cell death (BAD), BCL-2 interacting mediator of cell death (BIM) and BCL-2-associated X protein (BAX), with proteins that transmit pro-apoptotic signals, including the stress-responsive kinase (ASK1), forkhead box O1 (FOXO1) transcription factor and c-ABL kinase may connect *BCR-ABL1* TK signaling pathways to CML phenotype [34–39]. Here we showed that CBY1 binding with 14-3-3σ affects CBY1 expression in *BCR-ABL1*+ cells (both in the *C22orf2* K562 cell line, where CBY1 over-expression permits CBY1/14-3-3σ interaction, and in bone marrow cells from CML patients at clinical diagnosis). The central role of *BCR-ABL1* TK activity in CBY1 binding with 14-3-3σ is promoted by reduced CBY1 interaction with the scaffolding protein in response to IM (Fig 1B). A persistent increase in cytoplasmic and nuclear CBY1 associated with its IM-induced release from 14-3-3σ might be mostly mediated by enhanced transcription and cytoplasmic relocation (Fig 1A) [15,18–20]. AKT, a downstream target of *BCR-ABL1* TK, concurs to CBY1/14-3-3σ interaction through phosphorylation at serine 20 within the mode II-binding motif at CBY1 N-terminal region [17,23]. Accordingly, the persistent inactivation of AKT in response to the mTOR inhibitor RAD001 promotes the dissipation of CBY1/14-3-3σ complex both in the cytoplasm and nucleus of *C22orf2* K562 cells, hence preventing CBY1 nuclear export (Fig 3A and 3B) [17]. However, CBY1 expression in the cytoplasmic compartment was upraised following RAD001-induced inactivation of AKT (which prevents CBY1 phosphorylation at a critical residue for 14-3-3, 24 h IM treatment (which lets AKT re-activation) and 14-3-3 inhibitor BV02 (which neither affects *BCR-ABL1* TK nor AKT kinase activity), further supporting the impact of 14-3-3σ binding on CBY1 stability (Figs 1A and 1B, 2B and 3B, supplementary section S3 Fig) Notably, the dissociation from 14-3-3σ in response to IM, BV02 and RAD001 has antithetical effects on βcatenin expression (Figs 1A and 1B, 2B and 3C). The findings suggest that the interaction with 14-3-3σ, eventually mediated by *BCR-ABL1* TK-activity and AKT-induced phosphorylation of βcatenin at serine residues, protects βcatenin from degradation. Alternatively, the release and activation of GSK-3β from 14-3-3σ might contribute with GAS2 and Fap1 to address βcatenin degradation [12,13, 40].

A further regulatory mechanism of 14-3-3σ interaction with client proteins encompasses 14-3-3 post-transcriptional modifications occurring near the ligand-binding grove RSXpC/TXP and mediated by JNK phosphorylation. This mechanism is a pivotal regulator of cell response to genotoxic damage, proceeding from the release and nuclear targeting of c-ABL proto-oncogene [27]. Indeed, the *BCR-ABL1* TK blocks JNK-mediated phosphorylation of 14-3-3σ, hence precluding nuclear import and activation of the c-ABL protein encoded by the allele not involved in the rearrangement with BCR [41]. The cytoplasmic and nuclear CBY1 reduction in response to JNK inhibitor SP600125 is, indeed, associated with the persistence of CBY1/14-3-3σ complex, hence supporting that 14-3-3σ binding reduces CBY1 stability in the cytoplasmic compartment and concurrently precludes CBY1 nuclear import in a *BCR-ABL1*+ cell context (Fig 4A and 4B). To our surprise, JNK inhibition impaired βcatenin interaction with 14-3-3σ (Fig 4C). The mechanism(s) involved in βcatenin release from 14-3-3σ following JNK inhibition remain elusive. We can assume that SP600125 revokes JNK-induced phopshorylation of βcatenin at critical residues for 14-3-3 binding, while hindering 14-3-3σ phosphorylation at a critical residue for CBY1 binding [28,30]. Further investigation is required to elucidate the matter. In all instances, release from 14-3-3σ addresses βcatenin towards degradation, through events likely encompassing its enhanced degradation [12,13,42].

The role of JNK/14-3-3σ axis in CBY1 binding with the scaffolding protein and CBY1 reduction associated with *BCR-ABL1* TK was confirmed in the MCF from bone marrow samples of CML patients at clinical diagnosis. In such cell context, the reduction of CBY1 expression compared to the normal control (represented by MCF from the bone marrow of 8 HD pooled to avoid individual differences) was associated with CBY1 enhanced interaction with 14-3-3σ and reduced phosphorylation of JNK and 14-3-3σ at residues involved in the complex persistence in response to SP600125 (Figs 4 and 5).

Further investigation was addressed to establish whether UPS participated in CBY1 degradation. CBY1 increment in response to the proteasome inhibitor Bortezomib in *C22orf2* K562 cell line supports UPS role in CBY1 reduced stability associated with *BCR-ABL1* TK activity and binding with 14-3-3σ (Fig 6A). The identification of SUMO groups in the CBY1 sequence prompted us to investigate whether SUMOylation, a post-translational modification that utilizes SUMO groups to covalently attach target substrates and promote their ubiquitination and degradation, takes part in CBY1 increased degradation associated with *BCR-ABL1* TK (Takemaru et al, unpublished results). In *C22orf2* K562 cells CBY1 increment in response to drugs (IM, BV02 and RAD001, which promote CBY1 release from 14-3-3σ) was associated with CBY1 reduced SUMOylation, while CBY1 reduction in response to SP600125 (which let the persistence of CBY1 interaction with 14-3-3σ) was associated with CBY1 upraised SUMOylation (Fig 6B). Indeed, enhanced CBY1 SUMOylation distinguishes MCF from bone marrow samples of CML patients compared to normal control (Fig 6C). Notably, in such cell context, CBY1 expression and SUMOylation status were inversely correlated, with a decrease of CBY expression of more than 50% with respect to normal control and an almost 1,5-fold increment of CBY1 SUMOylation compared to the reference value (HD pool set to 1) (Fig 6D).

The crosstalk between SUMO and JNK signaling has been implicated in cell response to oxidative stress and inflammation. It pertains the homeodomain-interacting protein kinase 1 (HIPK1, whose de-SUMOylation promotes tumor necrosis factor α-induced activation of JNK), JNK-induced reduction of oxidant-enhanced SUMOylation of ataxin-1, SUMOylation impact on JNK activation under oxidative stress conditions and JNK-dependent SUMOylation of retinoid X receptor α in hepatocellular carcinoma [43–46]. Our results establish a putative link between *BCR-ABL1* TK activity-contingent enhancement of CBY1 SUMOylation and inactivation of JNK/14-3-3σ associated with the *BCR-ABL1* TK activity of CML. Further investigation is required to elucidate individual steps involved in SUMO and JNK crosstalk. A putative intermediate between the two might be GSK-3β, whose activation results in the attenuation of βcatenin signaling through mechanisms involving the binding with 14-3-3σ and JNK [41,47,48].

## Conclusions

Results shown here suggest that CBY1 interaction with 14-3-3σ is promoted by CBY1 post-transcriptional modification by the *BCR-ABL1* TK downstream target AKT. The interaction with 14-3-3σ reduces CBY1 stability in *BCR-ABL1*+ cells through events encompassing UPS and, in particular, protein SUMOylation. Our results open new routes in the search for molecular pathways promoting the proliferative advantage of leukemic hematopoiesis, particularly in the LSC compartment which is resistant to TK inhibitors.

## Supporting Information

S1 Fig 14-3-3σ expression in our experimental model: 14-3-3σ expression exhibits slight differences in in the cytoplasmic and nuclear compartments of *C22orf2* K562 cell line in response to drugs used to investigate differences in CBY1 expression relative to its interaction with 14-3-3σ. (PDF)

S2 Fig CBY1 transcriptional regulation after IM treatment: CBY1 increment in response to IM is driven by transcriptional mechanisms and conditional upon de-methylation of the *C22orf2* promoter. A-Both CBY1 transcript isoforms (340 bp and 200 bp) were raised since 1^st^ up to 5^th^ h of IM treatment. B-5mC and DNMT1 recruitment at *C22orf2* promoter were concurrently reduced in response to IM.
(PDF)

S3 Fig Drugs used to investigate CBY1 expression relative to its interaction with 14-3-3σ do not exhibit off target effects. (PDF)

S4 Fig CBY1 expression in the cytoplasmic and nuclear compartments of parental K562 cell line confirmed the results obtained in *C22orf2* K562 cell line. (PDF)

S1 Table (DOC)
