# Supplementary figures and images for: 14-3-3 Binding and Sumoylation Concur to the Down-Modulation of β-catenin Antagonist chibby 1 in Chronic Myeloid Leukemia
Source: PLoS One. 2015 Jul 6;10(7):e0131074. doi: 10.1371/journal.pone.0131074 (PMC4492953; doi:10.1371/journal.pone.0131074)

*C22orf2* K562

|          |   |   |   |   |   |
|----------|---|---|---|---|---|
| IM       | - | + | - | - | - |
| RAD 001  | - | - | + | - | - |
| BV 02    | - | - | - | + | - |
| SP600125 | - | - | - | - | + |

14-3-3 $\sigma$

ACTIN

14-3-3 $\sigma$

HISTONE H1

CYTOPLASM  
NUCLEUS

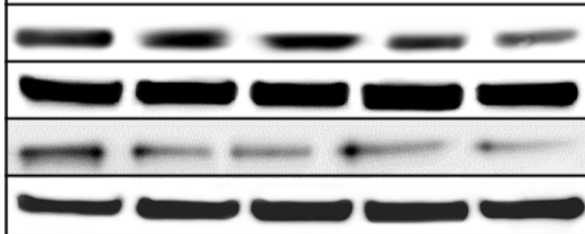

Supplement: S1 Fig — (PDF) [file pone.0131074.s001.pdf]

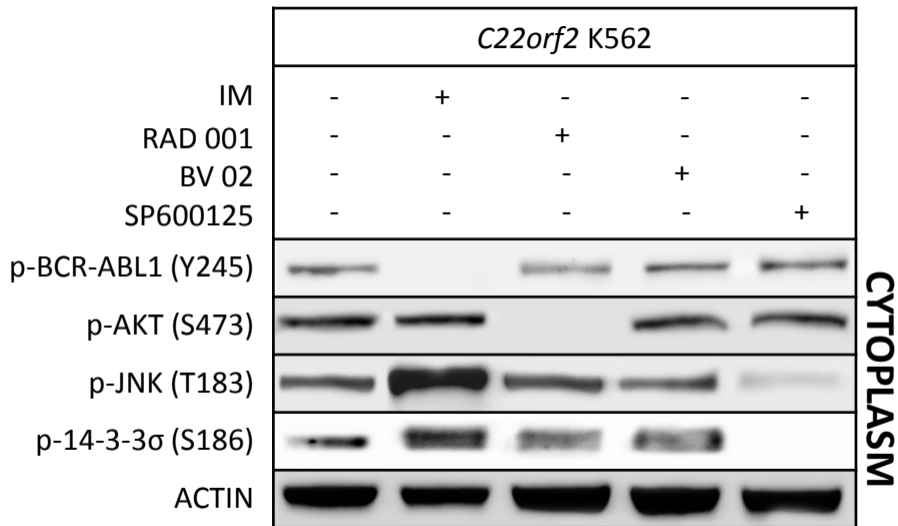

Supplement: S3 Fig — (PDF) [file pone.0131074.s003.pdf]

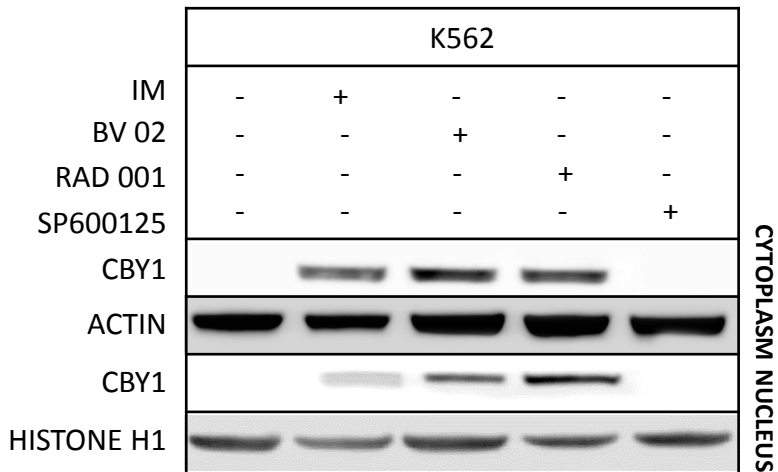

Supplement: S4 Fig — (PDF) [file pone.0131074.s004.pdf]
