# Supplementary material for: 14-3-3 Binding and Sumoylation Concur to the Down-Modulation of β-catenin Antagonist chibby 1 in Chronic Myeloid Leukemia
Source: PLoS One. 2015 Jul 6;10(7):e0131074. doi: 10.1371/journal.pone.0131074 (PMC4492953; doi:10.1371/journal.pone.0131074)
Supplement: S2 Fig — A-Both CBY1 transcript isoforms (340 bp and 200 bp) were raised since 1st up to 5th h of IM treatment. B-5mC and DNMT1 recruitment at C22orf2 promoter were concurrently reduced in response to IM. (PDF) [file pone.0131074.s002.pdf]

Figure S2

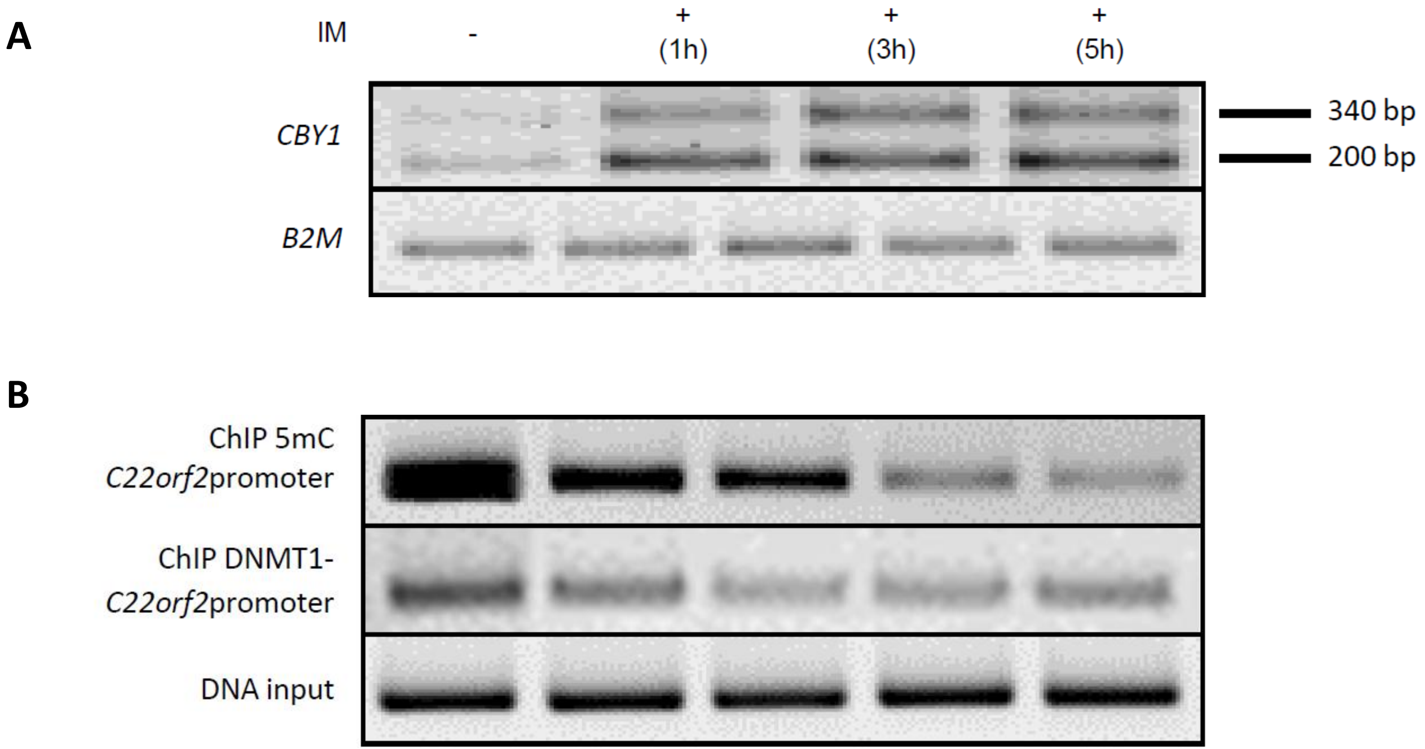
A
IM
-
+ (1h)
+ (3h)
+ (5h)
CBY1
B2M
340 bp
200 bp
B
ChIP 5mC C22orf2promoter
ChIP DNMT1- C22orf2promoter
DNA input
