# Supplementary material for: 14-3-3 Binding and Sumoylation Concur to the Down-Modulation of β-catenin Antagonist chibby 1 in Chronic Myeloid Leukemia
Source: PLoS One. 2015 Jul 6;10(7):e0131074. doi: 10.1371/journal.pone.0131074 (PMC4492953; doi:10.1371/journal.pone.0131074)
Supplement: S1 Table — (DOC) [file pone.0131074.s005.doc]

**Supplementary section**

**TABLE 1S**

| **PATIENT ID** | **SOKAL SCORE** | **TRANSLOCATION** | **CHR** | **MMR** |
| --- | --- | --- | --- | --- |
| PT1 | INT | t(9,22) | YES | YES |
| PT2 | INT | t(9,22) | YES | YES |
| PT3 | HIGH | t(9,22) | YES | YES |
| PT4 | INT | t(9,22) | YES | YES |
| PT5 | INT | t(9,22) | YES | YES |
| PT6 | HIGH | t(9,22) | YES | NO |
| PT7 | INT | t(9,22) | YES | YES |
| PT8 | LOW | t(9,22) | YES | NE |
| PT9 | LOW | t(9,22) | YES | NE |
| PT10 | LOW | t(9,22) | YES | YES |
| PT11 | INT | t(9,22) | YES | YES |
| PT12 | INT | t(9,22) | YES | NE |

The disease prognosis was based on the Sokal score at diagnosis and designated as low, intermediate or high risk of disease progression. Cytogenetic analysis performed at diagnosis underscored the type of translocation. All patients achieved a complete hematological response (CHR) at the 3^rd^ month of therapy with TK inhibitors. Eight patients achieved a MMR (3 log reduction of BCR-ABL1 transcript levels compared to diagnosis) within the 1^st^ year of therapy and the follow-up of remaining 3 patients was too short to evaluate the molecular response to therapy.
